# Women’s perception of cervical cancer pap smear screening

**DOI:** 10.1002/nop2.1196

**Published:** 2022-03-04

**Authors:** Kristine N. Siseho, Beauty Etinosa Omoruyi, Benjamin I. Okeleye, Vincent I. Okudoh, Hans J. Amukugo, Yapo G. Aboua

**Affiliations:** ^1^ 99404 Faculty of Science School of Nursing and Public Health University of Namibia Windhoek Namibia; ^2^ 70683 Applied Microbial and Health Biotechnology Institute Cape Peninsula University of Technology Bellville South Africa; ^3^ 70683 Department of Biotechnology and Consumer Science Cape Peninsula University of Technology Cape Town South Africa; ^4^ 59197 Department of Health Sciences Faculty of Health and Applied Sciences Namibia University of Science and Technology Windhoek Namibia

**Keywords:** cervical cancer, human papillomavirus, pap smear, reproductive age

## Abstract

**Aim:**

The study examines limiting factors associated with cervical cancer Pap smear screening among participants of reproductive age attending a healthcare facility in Namibia.

**Design:**

A cross‐sectional descriptive and exploratory study was conducted.

**Methods:**

The data were collected using a face‐to‐face interview (qualitative) and a structured questionnaire (quantitative). A total of 49 participants (10 qualitative and 39 quantitative) aged 17–45 years participated in the study.

**Results:**

The study revealed that 80% of participants have limited knowledge of cervical cancer, while 49% have never done the test before and 8% were not informed of the screening and risk of the disease. Furthermore, 49% of participants responded that the screening fees are not affordable. Meanwhile, all participants (100%) complained of the long waiting period. Other main barriers for not screening were missed announcements and unsuitable time allocation. Knowledge on cervical cancer and turn‐up for Pap smear screening test was low among participants of reproductive age.

## 1 INTRODUCTION

Cervical cancer is a sexually transmitted disease (STD) caused by the human papillomavirus (HPV), especially HPV–16/18. Over 1 million women worldwide are diagnosed with cervical cancer and approximately 90% of all death occurred in low‐ and middle‐income countries (Ferlay et al., 2018; Sung et  al., 2021; Taku et al., 2020). The disease is the eighth most common cancer among women from the age of 48 years. The rates of new cases or deaths have decreased by approximately 74% in the last 50 years, largely due to the widespread of Papanicolaou (Pap) smear screening and the introduction of HPV vaccines and treatment (Bruni et al., 2019). The National Health Science (NHS) implemented the Pap smear screening programme, which has been successful in the high‐risk age group of 25–49 years (every 3 years), and those ageing 50–64 (every 5 years). In Australia, it was reported that the number of women diagnosed with the disease has dropped by 90% due to the success of the testing programme and prevention, hence the prediction of recovering rate of 98% in 2028 (Bosch & Dreyer, 2017). According to Namibia National Cancer Registry (NNCR), cervical cancer is listed among the top three cancers in all ethnic groups ranging from 4.9% White female to 33% among Caprivian women (NNCR, 2017). The disease has become a major life threat among women between the ages of 15 and 44 years in Namibia, with over 132 new cases and 59 deaths recorded every year out of a population of 813,157 (Bruni et al., 2019; NNCR, 2017).

The common risk factor associated with cervical cancer is mainly the frequent non‐protected intercourse and risky sexual practices (oral sex) with multiple partners, who also may have other multiple sexual partners infected with sexually transmitted diseases, such as HPV (Lukac et al., 2018). The national guideline for family planning in Namibia revealed that HPV is found in semen and genital area and can be transmitted through oral sex, as an estimate of 50%–80% of sexually active women is infected with HPV, which can persist to cause pre‐cancerous growth (Husaiyin et al., 2018). It also noted that the use of continuous oral combined oral contraceptives (COC) for 5 years or more appears to speed up the development of persistent HPV infection, which may result in cervical cancer. Although the number of cervical cancers associated with COC use is thought to be very small, all COC users should be screened for any pre‐cancerous cells (Roura et al., 2016). Smoking is another important risk factor associated with cancer. It allows nicotine to be absorbed through the lungs and transported through the body by the bloodstream. Nicotine is then broken down and impair the immune system, and thus, the cancerous cells developed around the cervix. Moreover, some of the nicotine chemicals and by‐products have been found in the cervical mucus in women who smoke regularly (Kamiza, 2020). Frequent birth from the age of 35 years upwards, poor diet and lack of vitamins can also increase the risk of cervical cancer (Fontham et al., 2020; Koshiyama, 2019). A balanced diet with fruits and vegetables containing vitamins A, C and E can aid the body's natural defence against cancer and viral infections.

Cervical cancer can take up to 15–20 years to develop without signs or symptoms, and in later stages may result in abnormal vaginal bleeding, pelvic pain or pain during sexual intercourse and vaginal mass that may indicate the presence of malignancy (Sung  et al.,  2021; Mwaka et al., 2015). It is therefore important for women to have a regular Pap smear gynaecological examination for early detection and prevention as the cervical cancer stages arise from the tissue of the cervix and spread to the pelvis wall blocking the ureters and other nearby vital organs (Sarenac & Mikov, 2019). When abnormal results are detected in a patient, a more sensitive diagnostic examination using colposcopy is needed to examine the pre‐cancerous changes known as cervical intraepithelial neoplasia system (CIN) or squamous intraepithelial lesion (SIL) (Akinyemiju et al., 2015).

The current study focuses on the limiting factors associated with cervical screening among women attending a healthcare facility. This study seeks to assess the limiting factors associated with women's knowledge of cervical cancer and the perception about Pap smear screening and the provision of healthcare services. This study, therefore, investigated the vital components that played a major role in the quality of knowledge about healthcare services given by the healthcare facilities, such as awareness of the disease, accessibility of Pap smear screening, affordability, efficacy, test outcome management, prevention and general health improvement.

## 2 MATERIALS AND METHODS

### 2.1 Study design

A cross‐sectional descriptive and exploratory design was used in this study. The mixed method comprised of quantitative and qualitative techniques using a closed‐ended questionnaire to test the relationship between the variables and outcomes as well as an interview to understand the participants’ point of view about cervical cancer screening, respectively, were used.

### 2.2 Study area and population

This study was conducted at a healthcare centre in Namibia in collaboration with the Pap smear and breast examination clinic of an institution. The participants (women) between the ages of 17–45 years were targeted for the assessment of their limiting factors and provision about cervical cancer Pap smear screening service. Participants’ knowledge, perception, family history of cancer and affordability were determined. Women who visited the clinic in the period of the study were recruited by a Registered Nurse (RN) for participation after informed consent. Out of the 54 participants recruited in the study, 9.3% (5 potential participants) declined to participate.

### 2.3 Sampling method

Systematic random (the selection of samples at a pre‐set interval) and convenience sampling method was used to collect the data, while the sample size from the target population was calculated using the equation below and the questionnaire was distributed to every 6th person to collect the quantitative data (Sekaran & Bougie, 2016).

$$n=\frac{\alpha }{100}XN$$

Where:

*n* = sample size, *N* = total population (according to the statistics of the study area census in 2015, the population of the institute was 6,066) and *α* = confidence limit (4.5). The α value was determined based on the z value of 1.96 at the confidence level of 95% which is a normal population set constant, standard normal deviation (Kasiulevicius et al., 2006; Shivute et al., 2019). Of 273 participants, 10 were selected for interview (qualitative data) and 263 for questionnaire distributed to every 6th subject to collect quantitative data which is equal to 44 participants but 39 respondents as shown in Table 1. The sample‐to‐variable ratio has been suggested to be a minimum observation‐to‐variable ratio of 5:1; meanwhile, 15–20 observations (respondents or samples) per independent variable are strongly recommended of which this study has 3 variables (knowledge, perception and attitude), hence the number of the sample (Memon et al., 2020).

**TABLE 1:** Systematic sampling of the population

Systematic sampling	Sample description	Sample size
**Qualitative research**	Calculated sample size	273
	Interviewed (Qualitative)	10
**Quantitative research**	Questionnaire (Quantitative; selected every 6^th^ subject)	44 participants selected
	Invalidated questionnaire	1
	Questionnaire not returned	4
	Valid questionnaires	39 respondents
**Total**		49 participants

### 2.4 Data collection

Data for quantitative analysis were collected with a structured questionnaire distributed to 44 eligible participants who visited the clinic during the period of the study. The participants were given closed‐ended questions where they have to choose from the options given. Data for qualitative analysis were collected from 10 participants during a face‐to‐face interview conducted in a conducive environment at the clinic after informed consent. The participants were now given open‐ended questions to express themselves and their responses audio‐recorded for analysis. The data collected including demographic information were kept confidential and as anonymous as possible. The internal and external validity of the study was ensured by careful check of the questionnaires for correct language use and relevance and subsequently assessed by the peers to exclude errors. A pilot study was conducted to assess or confirm the accuracy of the data collected and the repeatability of the study.

### 2.5 Data analysis

The quantitative data were analysed using STATA 12.0 software. The pie chart, bar graph and tables were used to present the age distribution and general knowledge. Qualitative data were analysed using content analysis, where different themes and sub‐themes were highlighted and interpreted. The trustworthiness of analysis was ensured by conducting the study in a way that enhances the credibility of data so that there is an internal agreement between the investigators’ interpretation/findings and participants’ responses to the questionnaire. Hence, the repeatability, validity, dependability and accuracy of the data collection were ensured. The reliability tests were done to examine whether the questions were interpreted correctly or the ideas captured accurately. Misinterpretations were avoided between the researcher and the study by involving language interpreters during the study. Additionally, an interview guide was formulated to aid in ensuring that all information required for the study was collected. Moreover, the dependability of the study was achieved by giving a period of 5–10 min to each participant to ask questions and to express their opinion.

### 2.6 Ethical considerations

Ethical clearance was obtained from the University, and Ministry of Health and Social Services (MoHSS) and the School of Nursing (Ref.: 17/3/3 KS). An informed consent form, along with a well‐structured questionnaire, was distributed to all participants, and the individual interviews were conducted in a private room at the clinic. Privacy was maintained throughout the study with no names recorded on the questionnaires, and individual confidentiality was respected at all costs. The participants were not coerced to enrol in the study as the right to refuse was explained without any negative impact on their medical care. Ethical consideration was respected at all costs about issues of confidentiality, honesty and free participation in the study.

## 3 RESULTS

### 3.1 Analysis of the participants

Overall 54 women enrolled to participate in this study. One of the questionnaires was considered invalid due to failure to answer some of the questions, while 4 participants did not return the questionnaire at all, hence a total of 49 participants were analysed (Table 1). Of all the participants, those from age 17–25 years were 46%, the larger part of the respondents who represent the undergraduate students compared to 31% (26–35 years) and 23% (36–45 years) of those who represent the postgraduate students and working class adults (Table 2). Considering the marital status, this explains the reason for the highest number of 23 (59%) single respondents, when compared to 14 (36%) married, 1 (2%) divorcees and 3% widowed respectively (Table 2). To assess the affordability of the service, the employment status of all participants was recorded, with a total number of 13 (67%) and 26 (33%) participants who were unemployed and employed respectively (Table 2).

**TABLE 2:** Demographic analysis

Age group (%)		Marital status (%)		Employment (%)	
17–25	46	Single	59	Yes	33
26–35	31	Married	36	No	67
36–45	23	Divorced	2	–	–
–	–	Widow	3	–	–

### 3.2 Factors that impact pap smear screening

**FIGURE 1:**
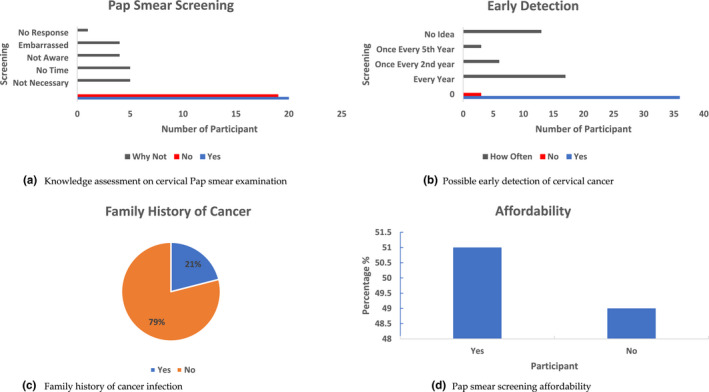
Women's perception and the influence of money, family history and knowledge. (a). Knowledge assessment on cervical Pap smear examination. (b). Possible early detection of cervical cancer. (c). Family history of cancer infection (d). Pap smear screening affordability

When participants were asked questions such as, “Have you done Pap smear before?” to assess the participant's general knowledge about the Pap smear service provision. Exactly 51% (20) confirmed their voluntary screening, while 49% (19) mainly among the participants from age 17–25 years have never done the screening before as shown in Figure 1a. Further sub‐question “why” was asked to assess the depth of knowledge about cervical screening from those participants who answered “no” (never done Pap smear). The reply showed that 26% (5) of the participants that have never screened before did not see the necessity of doing the test, meanwhile, 26% (5) responded that they had no time to do the screening. Furthermore, 21% (4) were not aware as nobody informed them about pap smear screening and 21% (4) with a feeling of embarrassment coming forth because the test is done in their private parts (Figure 1a). There was no response from 1 (5%) participant. When asked if it is possible for cervical cancer to be detected early, of the 39 respondents 36 (92%) were informed of the possible early detection of cervical cancer through the Pap smear screening and 3 (8%) participants responded otherwise. Moreover, further information on how often should the Pap smear examination be done revealed that 44% (17) of the participants were informed or assumed that the screening should be done every year, 15% (6) once every second year and 8% (3) once every fifth year, while 33% (13) have no idea as shown in Figure 1b. Family history of cancer infection, which is one of the predisposing risk factors for individuals to develop cervical cancer was assessed. The study revealed that 8 of the participants have a family member who has cancer, while 31 replied otherwise (Figure 1c). The affordability of the Pap smear screening among the participants showed that with the service fee of N $50.00 (USA $3.40) and N $70.00 (USA $4.76) for students and working class members, respectively, 20 (51%) respondents indicated that the service fees for both students and working class is affordable, meanwhile, 19 (49%) participants responded that the fees are not affordable (Figure 1d).

### 3.3 Analysis of the interview

Ten (10) participants were interviewed with 6 (60%) from age categories of 17–25, 2 (20%) of 26–35 and 2 (20%) of 36–45 years, and their response is represented in Table 3. The responses were branded into themes and sub‐themes covering the knowledge of cervical cancer examination, the importance of the screening and the condition of services were given at the facility. The findings show that about 8 (80%) out of the 10 participants, most of the 17–25 years age group have limited understanding and knowledge on cervical cancer screening and its risk factors. In addition, the participants have some misconceptions about the purpose and procedure of Pap smear. The lack of knowledge is evident from the following narratives as statements and questions: “Is it the process where women undergo an examination of the cervix to detect any signs of cancer of the cervix?” “The test is for cleaning the womb to remove impurities,” “A rod is passed through the vaginal into your womb and it's very painful,” and “The test is to cure cancer and womb infections” As well as “What I knew is that the doctor inserts a knife and cut a small piece of flesh and then test it, but today the nurse who was teaching us said there is neither knife nor cutting, they just take white things and is not painful at all.”

**TABLE 3:** Qualitative summary of the interview

Themes	Sub‐theme	Meaning unit
1. Understanding of cervical cancer screening.	1.1. Knowledgeable or lack of Pap smear screening.	Pap smear is used to clean the womb, remove infections and increase the chance of getting pregnant.
		Pap smear must be something they put inside to clean private parts.
		Never heard about Pap smear.
		Not sure what Pap smear is.
	1.2. Lack of knowledge for developing cervical cancer.	I just know that if you are having unprotected sex then you are at risk.
		No need to screen for cervical cancer if one feels fine.
	1.3. Misconceptions about Pap smear screening	Pap smear removes infections in the womb.
		Pap smear is the same as a biopsy.
	1.4. Lack of understanding of the procedure	Pap smear same as dilation and curettage. A piece of flesh will be cut during Pap smear.
2. Necessity of cervical cancer screening	2.1. Low or high perception of risk involved	A family member died of cervical cancer.
3. Service delivery at the facility	3.1. Poor service delivery	Long waiting times about service and follow‐up for Pap smear result.

The results also show that all participants are not aware of the seriousness of the risk of developing the disease, or said that there is no need to go for screening if there are no development of signs and symptoms of infection, as related: “There is no cause for alarm I think am fine, it's not a serious issue to be worried about,” “Can having sex with different men, put women at risk of having the disease? I only know of HIV.” Some believe that the purpose of the Pap smear test is for those that have existing cervical cancer disease, therefore, the necessity of going for screening is not important when one does not have signs or symptoms.

In this study, about 20% of the participants understood that one of the reasons for doing Pap cervical cancer screening is due to family history of the disease, as narrated: “Yes my mother passed away due to cervical cancer and being a woman and mother, chances of having the disease maybe 50%,” “Yes the test is necessary, especially at my age (36) to know my status about the infection.” Other participants due to ignorance or because of the perception that the risk of contracting the disease is statistically insignificant, and as such did not see it necessary to do the screening as related: “Not yet because I never consider it, maybe next year.”

Meanwhile, all participants complained of a long waiting time before the examination and result collection, due to just one qualified Registered Nurse (RN) that is capable of the test. The other RN is busy with the administrative paperwork. They spotted that the long queues discourage people that come for the test. Poor service delivery can be a major barrier for the large uptake of participants’ screening, has narrated: “The problem is that when you come for the test, you may end up waiting the whole day without being assisted,” “Even to collect your results, you are delayed unnecessarily or asked to wait to see the doctor” and “It is better to go to a private doctor for the test, just that the fee charges are much higher than Government clinics.” The participants responded that there is a need to address some operational issues which include that the specified dates are given to patient's test examination and collection, few numbers of patients per/day to reduce the waiting time, and internal referrals. It was also stated that more RN should be involved in the screening test, and good services should be rendered in a manner that increases the accessibility of cervical cancer screening.

## 4 DISCUSSION

This study revealed that 49% of the participants mainly from age 17–25 years (Table 2) have never participated in Pap smear screening before as shown in Figure 1a, the reason being that some have not heard about the screening or did not see the necessity to go for it. The study conducted by Saha et al. (2017), reported similar factors among young women from 19–29 years not showing interest in the uptake of Pap smear cervical cancer screening when compared to the older age women. However, the study reported that the underexposed to screening among the younger age group might not pose a statistically significant public health concern in the short and medium term (5–10 years), as they are less probably develop cervical cancer compared with the older women aged 30–50 years. Nevertheless, it is essential to target the younger age group as they are most sexually active and hence can become a victim of the disease than can be asymptomatic and take up to 20 years to fully develop into cervical cancer. According to CDC, if the result is HPV negative, the doctor will probably suggest a waiting period of 3 years before the next test. This applies to participants between the ages of 30–65 years who might be advised to do a combined Pap smear and human papillomavirus (HPV) test. But for participants over 65 years, may no longer be necessary to do the screening except if they have previously been treated for a pre‐cancerous cervical lesion or cervical cancer, living with HIV, or exposed to diethylstilboestrol before birth. The perception of women of the reproductive age in this study however demonstrated some knowledge about the importance of cervical cancer examination.

About service provision, the RN nurses at the clinic have shown good competence in performing cervical Pap smear screening for both students and the working class. Furthermore, the study revealed that adequate and efficient service delivery to the people coming in large numbers for the screening can be hindered by a few numbers of RNs available at the clinic. The study also noted that the institution does re‐assign time and dates for all activities and appointments, and this can also hinder the service provision of cervical screening for patients who may not find the postponement convenient for them. Women's change in scheduled appointments has been noted as a problem by another research study, for example, a study conducted by Baranoski et al. (2011) showed that re‐appointments have been one of the major factors influencing the cervical screening behaviour of women in Zimbabwe. Extended waiting periods at the health clinic has been observed in this study which is similar to the reports from other studies and the diverse approach of health workers towards patients which are constraints that influence low turn‐up of patients for Pap screening test, which may result in women not being screened for life, even if they have the intention (Phil et al., 2018).

Our finding as shown in Table 3 is also in line with the study conducted by Ali‐Rasasi et al. (2014) in Kinshasa, Democratic Republic of Congo (DRC) who reported a lack of understanding and knowledge on the early detection of cervical cancer Pap smear among the women. Moreover, the lack of understanding of the procedure of vaginal examination has also been a barrier to cervical cancer screening as women develop a fear of the screening procedure (Saha et al., 2017). The majority of women's perception in Nigeria on the risk of having cervical cancer has been that the purpose of the Pap smear test is for those that have existing cervical cancer disease, therefore, the necessity of going for Pap screening is not important when one does not have signs or symptoms (Ndikom & Ofi, 2012). Similar to the response in our current study, the qualitative study conducted among Malaysian Zimbabwean women, revealed that their misconceptions about the cervical screening test are that it is used for cleaning the womb and treating infertility (Chipfuwa & Gundani, 2013). Intensive knowledge and accurate information about the purpose of Pap smear screening should be appropriately interpreted during community engagement or individual discussion that screening is primarily used to detect precursor lesions that occur early in the course of the disease for early treatment.

Major limitations noted were long queues, long waiting times, service charges (Figure 1d), and misconceptions (Table 3). Some participants responded that they never had time to go to the clinic for the test because they are always at work or very busy with schoolwork. This corroborates the other report which noted that long working hours appear to prevent women from attempting to attend screening sites for the test (Barroilhet et al., 2013). Across all the focus groups, the thought of being embarrassed, fear of the outcome, and the process of doing a Pap smear, makes most of the participants uncomfortable and hence influenced their behaviour towards the test. Participants were also worried about the stigma that might be attached to them by friends if they attended the clinic for Pap smear screening, not knowing that every health issues are highly confidential between the individual and the nurse or doctors (Binka et al., 2019; Dodd et al., 2020).

It is therefore recommended that appropriate service provisions should be made readily available on‐demand so that both students and workin class will not develop negative behavioural attitudes towards testing. Availability of service delivery will improve the intake of cervical cancer examinations and thus contribute to the women's total wellbeing and confidence. The unemployment status of the participants at 67% as shown in Table 2 revealed the main reason for 49% (Figure 1d) not being able to afford the screening fees. The married participants (Table 2) may also have better financial support to afford the screening than the others. The service fees should be made free or be reduced to at least 10–20 N$ especially for students who may not be able to afford it (Figure 1d). Nevertheless, both students and working class should be informed that the service fee collection is donated to the Cancer Association of Namibia (CAN) to assist those who are striving with cancer disease.

This study only focused on women of the reproductive age group of 17–45 years in an institution in Namibia, hence the data cannot be generalized to the entire population. Another limitation was the willingness of all the selected people to respond to the questionnaire. This could have helped to achieve a better result. However, since only one respondent filled the questionnaire incorrectly, it had little impact on the study and thus was quickly excluded, while the 4 participants that did not return the questionnaire have no data contribution. Access to health care should be made easily available for every woman to ensure cervical cancer screening provision. Service delivery should also be intensified by raising the awareness of the disease and the early prevention in the communities and on campuses through media and other social devices. Publicizing the screening test via radio and television broadcasts will help to facilitate the spread of the information, especially allowing a witness who has undergone a Pap screening test to testify.

For capacity building, the Department of Health Services should liaise with other stakeholders such as the MoHSS, and CAN to consider purchasing cervical cancer screening equipment and distribute them to various health facilities for free or at a subsidized fee for screening. They should also conduct workshops in public places and institutions to address the misconceptions and fear about cervical screening activities, which have been shown to influence the uptake of cervical cancer screening mobility. Women living with HIV and those with multiple partners, along with smoking, heavy drinking and unhealthy eating, should be informed of the high risk of developing the disease. Regular surveillance is therefore encouraged to monitor and analyse the effects of these interventions in future studies.

## 5 CONCLUSIONS

The study contributes to the awareness of cervical cancer and factors limiting the uptake of Pap smear screening. Findings revealed that the participants have acknowledged the importance of Pap smear screening for women's general wellbeing. The participants who never did Pap smear tests before, was due to lack of knowledge, though some indicated that they cannot afford the service fee and as such hindered their screening participation. However, having participated in the study has raised their interest and confidence to go for the screening. An increase in awareness creation (counselling and workshops), a free Pap smear screening available on‐demand, or a subsidized rate will help to improve the regular screening compliance.

## CONFLICTS OF INTEREST

All the authors report no conflict of interest related to this manuscript.

## AUTHOR CONTRIBUTION

All authors meet the authorship criteria, and all authors agree with the content of the manuscript: K.N.S, B.I.O, Y.G.A and H.J.A: methodology. K.N.S: investigation and original. K.N.S, B.I.O, B.E.O, V.I.O, Y.G.A, and H.J.A: resources. Y.G.A, H.J.A, B.I.O, B.E.O, and V.I.O: draft, editing, and review. H.J.A and Y.G.A: Supervision, project administration and conceptualization. All authors approved the final version for submission and publication.

## Data Availability

Data Availability Statement: The data that support the findings of this study are available on request from the corresponding author. The data are not publicly available due to privacy or ethical restrictions.
